# Transplantation of olfactory ensheathing cells promotes the therapeutic effect of neural stem cells on spinal cord injury by inhibiting necrioptosis

**DOI:** 10.18632/aging.202758

**Published:** 2021-03-04

**Authors:** Xiaoyu Wang, Naifeng Kuang, Yuexia Chen, Guifeng Liu, Nan Wang, Fan’er Kong, Shouwei Yue, Zuncheng Zheng

**Affiliations:** 1 Rehabilitation Center, Qilu Hospital, Cheeloo College of Medicine, Shandong University, Jinan 250012, Shandong, China; 2 Department of Rehabilitation, Taian City Central Hospital, Taian 271000, Shandong, China; 3 Shandong First Medical University and Shandong Academy of Medical Science, Taian 271000, Shandong, China

**Keywords:** cell transplantation, olfactory ensheathing cells, neural stem cell, necrioptosis, spinal cord injury

## Abstract

Transplantation of neural stem cells (NSCs) is one of the most promising treatments for spinal cord injury (SCI). However, the limited survival of transplanted NSCs reduces their therapeutic effects. The aim of the present study was to examine whether a co-transplantation of olfactory ensheathing cells (OECs) may enhance the survival of NSCs and improve the beneficial effects of NSCs in rats with SCI, as well as to investigate potential mechanisms underlying such efficacies. Co-transplantation of OECs and NSCs was used to treat rats with SCI. Sympathetic nerve function was determined by measuring sympathetic skin responses. The results showed that OEC/NSC co-transplantation improved motor function and autonomic nerve function in rats with SCI. Co-transplantation of OECs promoted NSC-induced neuroprotection and inhibited programmed necrosis of NSCs, which was mediated by receptor-interacting protein kinase 3 (RIP3) and mixed lineage kinase domain-like protein (MLKL). Furthermore, OECs increased the proliferation and differentiation of NSCs *in vitro*, and improved the survival rate of NSCs *in vivo*. Taken together, we conclude that transplantation of OECs inhibited programmed necrosis of co-transplanted NSCs to promote therapeutic effects on SCI. Therefore, co-transplantation of OECs and NSCs may represent a promising strategy for treating patients with SCI.

## INTRODUCTION

Recently, the incidence of spinal cord injury (SCI) has been increasing each year. Currently, there are more than three million patients with SCI in the United States, and the rate is currently increasing by 17,000 individuals per year [1]. In recent years, emerging data have suggested that cell-transplantation therapies represent a potentially effective therapeutic intervention for SCI by repairing and regenerating injured neurons [2, 3]. There are currently many candidate cell types for transplantation, among which neural stem cells (NSCs) and olfactory ensheathing cells (OECs) are two of the most promising cell types [4].

Despite recent progress in promoting regeneration of the spinal cord through transplantation of different NSC types after SCI [5], the degree of functional recovery has been limited. There are still many factors that influence the effects of NSC transplantation. One of these parameters is the hostile microenvironment of the injured spinal cord, which is not permissive for grafted NSCs [6]. After SCI, ischemia, hypoxia, and accumulation of inflammatory factors in the local microenvironment lead to the death of transplanted cells. Programmed necrosis, also known as necroptosis, has recently been identified as another type of programmed cell death. In particular, necroptosis is involved in the pathophysiology of diseases and trauma within the central nervous system and can occur in various cells within damaged neural tissue after SCI, including neurons, astrocytes, and oligodendrocytes [7]. In the current study, we demonstrated that receptor-interacting protein kinase 3 (RIP3) and mixed lineage kinase domain-like protein (MLKL), which are critical mediators of necroptosis [8], were upregulated in the SCI microenvironment and reduced the survival rate of transplanted cells. In order to solve this problem and improve the therapeutic effects of transplanted NSCs, a feasible strategy may be to select a neuroprotective cell and transplant it in combination with NSCs.

Our previous studies have shown that transplantation of OECs can promote sensorimotor and autonomic nerve recovery and reduce neuropathic pain after SCI [9, 10]. OECs, a special type of glial cell, exhibit characteristics of both astrocytes and Schwann cells and are capable of secreting large amounts of neurotrophic factors [11, 12]. These characteristics have been shown to help establish an amenable microenvironment [13–15] for the growth of injured axons and for promoting axons to reach their targets [16, 17]. Multiple studies have suggested that co-transplantation of NSCs and OECs may exert synergistic effects in promoting neural regeneration and improving recovery of locomotor function [18, 19]. Since this strategy has potential to improve the SCI microenvironment and yield improved therapeutic effects, we hypothesized that OECs may support the survival of NSCs and enhance the efficacy of NSC transplantation in the treatment of SCI.

In the present study, we investigated the effects of co-transplantation of NSCs and OECs on sympathetic function following SCI and used sympathetic skin responses (SSRs) to assess sympathetic neural function in Sprague-Dawley rats. Collectively, our findings reveal novel strategies for co-transplantation of NSCs and OECs to ameliorate SCI and elucidate its underlying mechanisms.

## RESULTS

### Culturing and identification of OECs and NSCs

**Figure 1:**
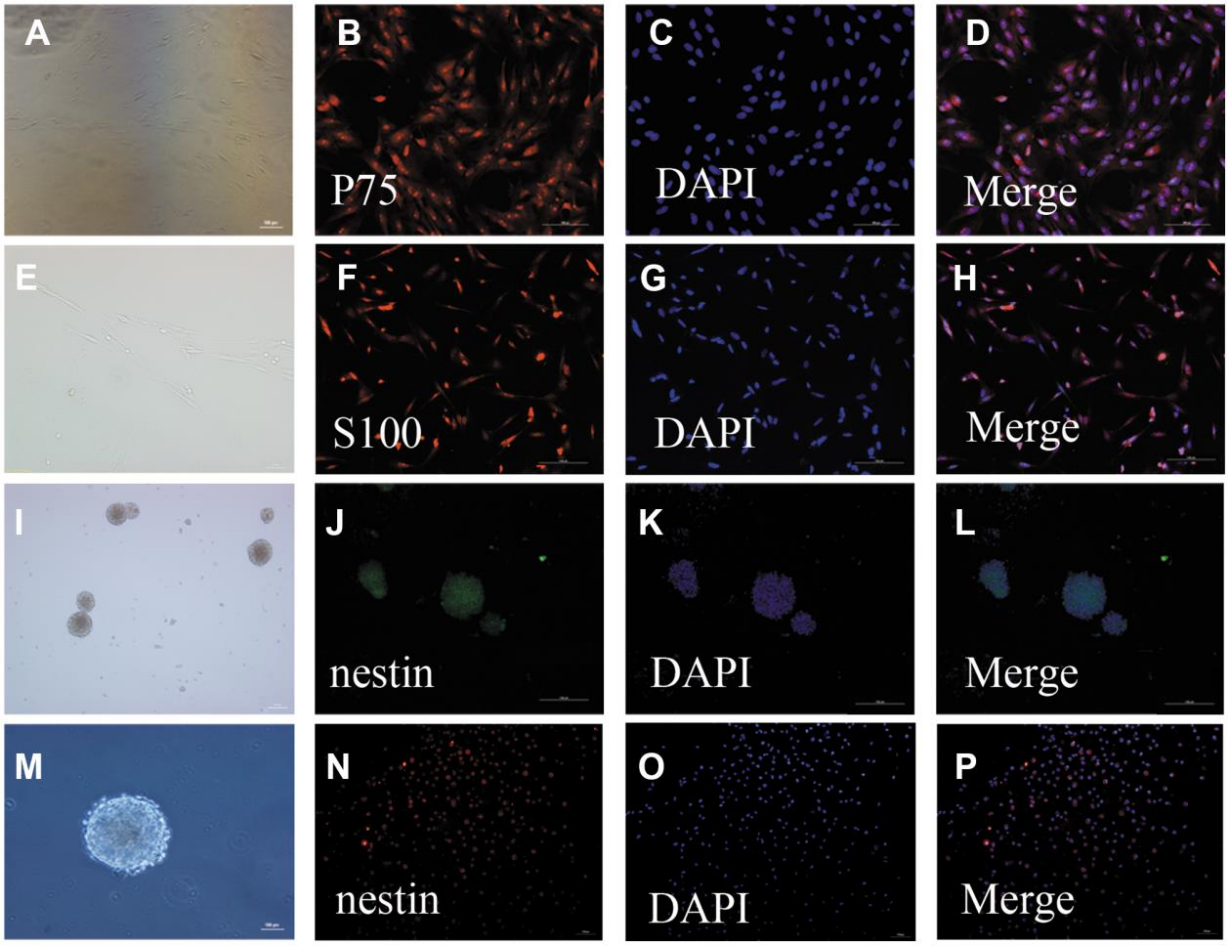
**Identification of OECs and NSCs:** (**A**–**H**): Phenotypic characterization of OECs: The appearance of OECs observed by phase-contrast microscopy (a: scale bar: 100 μm, e: scale bar: 50 μm). Immunofluorescent staining showed P75-positive cells (**B**–**D**): scale bar: 100 μm) and S100-positive cells (**F**–**H**): scale bar: 100 μm), which demonstrated that these cells were OECs. (**I**–**P**): Phenotypic characterization of NSCs: The appearance of NSCs observed by phase-contrast microscopy (i: scale bar: 100 μm, m: scale bar: 50 μm). Immunofluorescent staining of NSCs using nestin antibody (**F**–**H**): scale bar: 100 μm). The neurospheres were positive for nestin, suggesting that they were NSCs. Single-cell immunofluorescent staining of NSCs expressing nestin (**N**–**H**): scale bar: 100 μm). The purity of NSC cells was evaluated by the percentages of nestin-positive cells and DAPI-positive cells.

After 24 h in culture, single cells were round, transparent, and adherent; proliferating cells were observed after 48 h in culture. The cells were mainly bipolar and multipolar, with large cell bodies and oblong nuclei. After 5–7 days in culture, the number of OECs was increased and the cell bodies had many protuberances. Additionally, their protuberances became longer and all cells assumed clone-like growth, during which they spread to the periphery. After 12 days in culture, the cell density continued to increase and was arranged in a palisade (Figure 1A, 1E). Immunofluorescent staining showed that the OECs were double-positive for nerve growth factor receptor p75 (P75) (Figure 1B–1D) and S100 beta (Figure 1F–1H).

Primary NSCs were cultured for seven days, after which the cells were aggregated into suspended spheres for growth. These cells had a regular spherical shape with a dark center and bright edges (Figure 1I, 1M). Immunofluorescent staining of these cells was then performed using a nestin antibody. The neurospheres were positive for nestin, suggesting that they were NSCs (Figure 1J–1L, 1N–1P).

### OECs promote NSC survival in the SCI microenvironment

**Figure 2:**
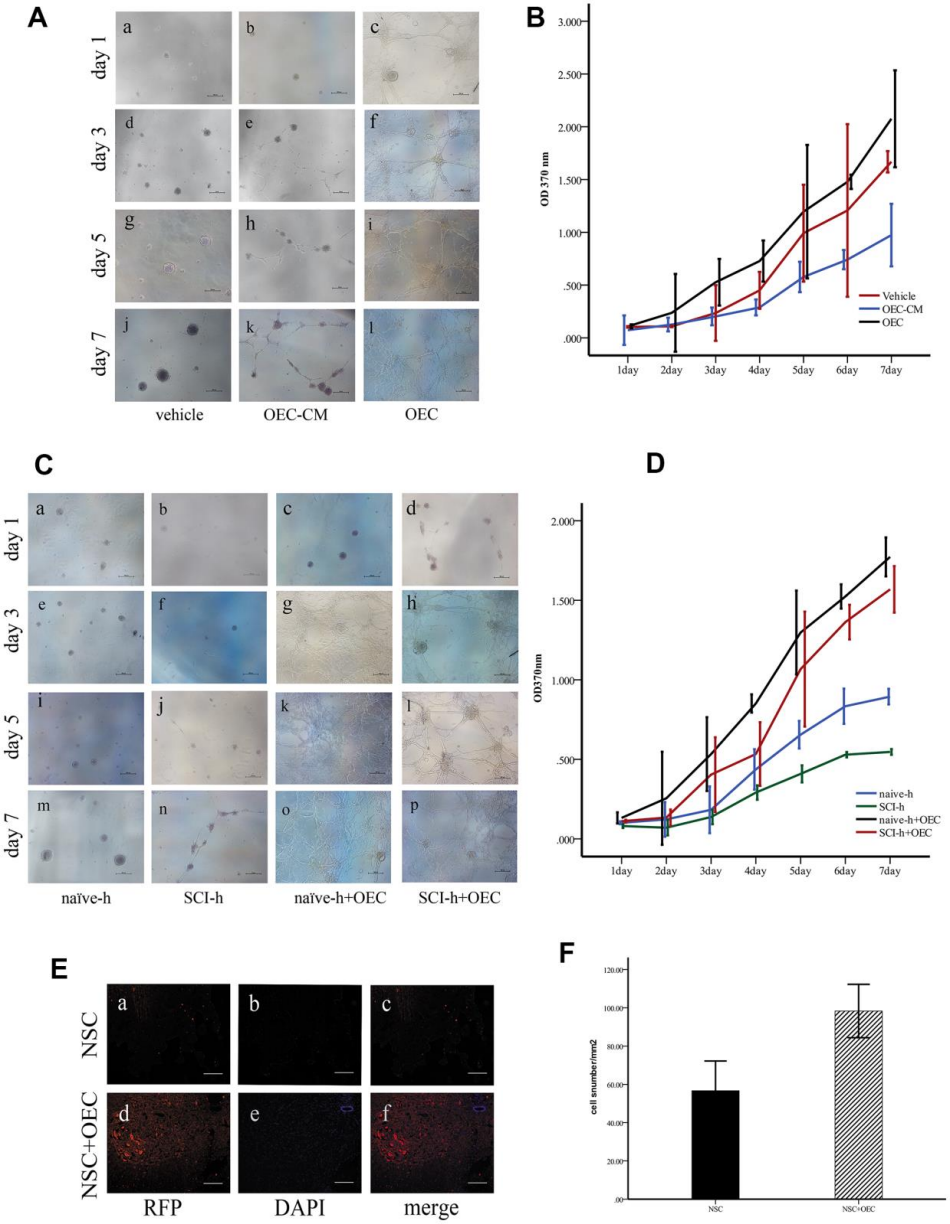
**OECs promote NSC survival in the SCI microenvironment.** (**A**) *In vitro*, the morphologies of the cells were observed under an inverted microscope. Scale bar: 100 μm; (**B**) The proliferation rates of NSCs assessed via MTT assays (mean ± SEM; two-way ANOVA, error bar: 95% CI). (**C**) Effects of OECs on NSC proliferation under SCI conditions observed under an inverted microscope. Scale bar: 100 μm; (**D**) The proliferation rates of NSCs assessed via MTT assays (mean ± SEM; two-way ANOVA, error bar: 95% CI). (**E**) *In vivo*, the survival of NSCs in different groups: (**A**–**C**) NSC transplantation alone, where RFP-positive cells were NSCs. Scale bar: 100 μm. (**D**–**F**) Transplantation of NSCs and OECs. Scale bar: 100 μm. (**F**) Quantification of the survival of RFP^+^ cells at two weeks after transplantation (means ± SEM, n=6; P<0.05, one-way ANOVA).

First, to investigate the effect of OECs on NSC proliferation, we cultured NSCs with OEC serum-free conditioned medium (OEC-CM) or OEC suspensions to observe their growth; for the vehicle group, complete culture medium of NSCs was used. Proliferation levels were measured by MTT assays. The results showed that either OEC suspensions or OEC-CM significantly promoted increased NSC proliferation compared with that of the vehicle group (P < 0.01). Moreover, the effect of OEC suspensions on NSC proliferation was significantly greater than that of OEC-CM (P < 0.01) (Figure 2A, 2B).

Next, we investigated the effects of the SCI microenvironment on the survival of NSCs and whether OECs could improve NSC survival in this microenvironment. NSC cultures were treated with homogenate (100 μg/mL) from the injured (SCI-h) or naïve (naïve-h) spinal cord and OECs (SCI-h+OECs or naïve-h+OECs) were also added to these cultures for seven days. The morphologies of the cells were observed under an inverted microscope every other day (Figure 2C). We found that NSCs cultured with naïve-h homogenate aggregated into suspended cellular spheres for growth. After seven days in culture, both the number and volume of cellular spheres increased, but the cells did not differentiate. However, the proliferation of NSCs in the SCI-h group was slowed compared to that in the naïve-h group, and the number and diameter of clone pellets were not significantly increased after five days of culture. After co-culturing with OECs for one day, all the cells adhered to the wall and formed a network with long protuberances, and this continued until the fifth day. MTT assays showed that NSC proliferation in the SCI-h group was significantly inhibited compared with that in the naïve-h group; in contrast, the number of cells in the SCI-h+OEC group was increased significantly compared with that of the other groups (Figure 2D).

Furthermore, we observed and quantified the survival of transplanted NSCs *in vivo*. We infected NSCs with a lentivirus labeled with red fluorescent protein (RFP) and transplanted them into the spinal cord of T10 SCI rats. The results showed that the number of RFP-positive cells in the OEC+NSC group was increased and the average area of RFP-positive cells was larger than that in the NSC group (Figure 2E, 2F).

### OECs inhibit RIP3/MLKL-mediated necroptosis of NSCs

**Figure 3:**
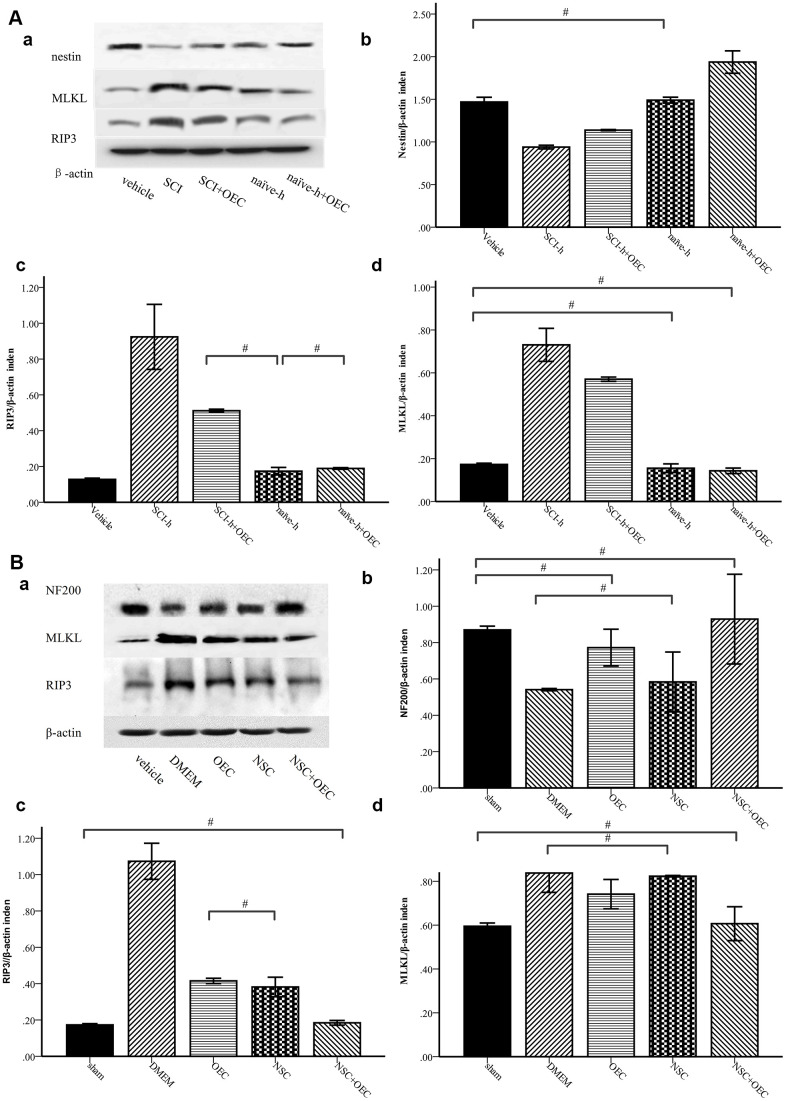
**Western-blot analysis reveals that OECs downregulate the expression of RIP3 and MLKL and inhibit SCI-induced increases of RIP3 and MLKL.** (**A**) *In-vitro* Western-blotting analysis showed that SCI induced upregulated expression of RIP3 and MLKL, and that OECs counteracted these changes to protect NSCs. (**a**) The protein expression level evaluated via Western blotting. (**b**–**d**) Data presented are expressed as the mean ± SEM (n = 6; one-way ANOVA, *post-hoc* test: Dunn-Bonferroni; error bar: 95% CI; ^#^P>0.05 and except for # in the figure; P values were all less than 0.05). (**B**) Two weeks after transplantation, protein levels of NF200, RIP3, and MLKL showed that OECs suppressed RIP3/MLKL-mediated necroptosis and protected NSCs. (**a**) The expression levels of NF200, RIP3, and MLKL were detected by Western blotting. (**b**–**d**) Data presented are expressed as the mean ± SEM (n = 6; one-way ANOVA, *post-hoc* test: Dunn-Bonferroni; error bar: 95% CI; ^#^P>0.05, except for the # in the figure; P values were all less than 0.05).

To determine whether OECs can inhibit necroptosis of NSCs induced by the SCI microenvironment, we treated NSCs with SCI-h or naïve-h, as well as with OECs, for seven days. Protein levels of nestin, MLKL, and RIP3 were assessed via Western blotting. As shown in Figure 3A, compared with those in the naïve-h group, RIP3 and MLKL levels were increased while nestin was reduced in the SCI-h group, which indicated activation of necroptosis in the SCI microenvironment and death of NSCs. Moreover, the expression levels of RIP3 and MLKL were significantly down-regulated when NSCs were treated with OECs (Figure 3A). To further elucidate changes in NSCs within the spinal cord following SCI, we transplanted OECs and/or NSCs into SCI rats and subsequently measured protein levels of RIP3, MLKL, and neurofilament 200 (NF200) via Western blotting (Figure 3B). Two weeks after transplantation, RIP3 and MLKL protein levels in the control group (i.e., incubated only in Dulbecco’s modified eagle medium [DMEM]) were still increased, indicating that necroptosis continued beyond two weeks after injury. Compared with those in the DMEM group and NSC group, the expression levels of RIP3 and MLKL were significantly lower in the OEC group and OEC+NSC group, which showed that OEC transplantation inhibited SCI-induced necroptosis. Compared with that in the NSC group, NF200 expression was significantly increased in the OEC+NSC group, suggesting that OECs played a neuroprotective role by inhibiting RIP3/MLKL-mediated necroptosis.

### Morphological changes in SCI rats after cell transplantation

**Figure 4:**
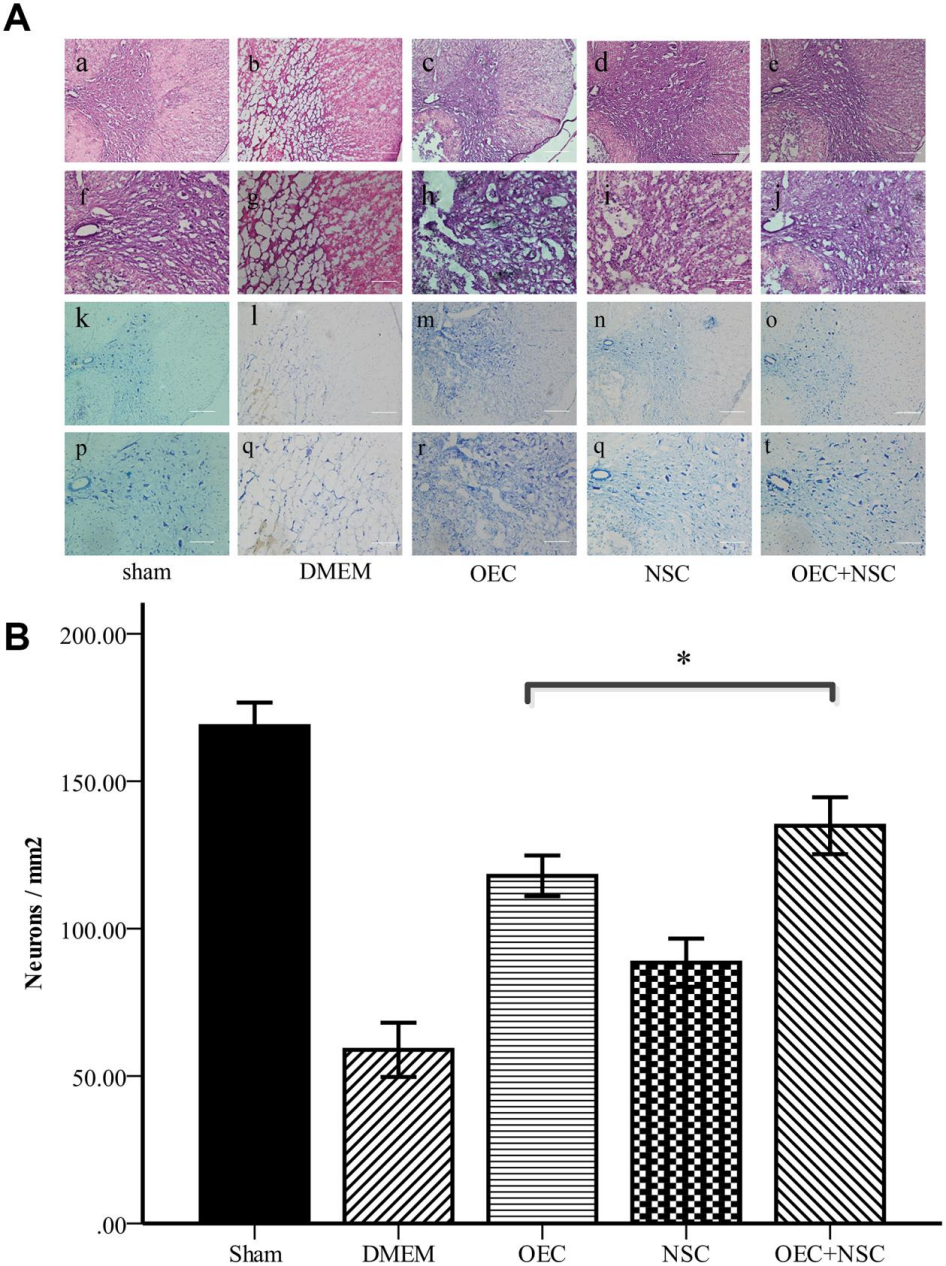
(**A**) Morphological changes at one week after SCI. (**a**–**j**) HE staining in each group. Scale bars: **a**–**c** 100 μm, **f**–**j** 50 μm. (**k**–**t**) Nissl staining in each group. Scale bars: **k**–**o** 100 μm, p–t 50 μm; (**B**) Quantitative analysis showed that the number of neurons in the OEC+NSC group was significantly increased in comparison with that in the DMEM group or NSC group. (mean ± SEM; one-way ANOVA, *post-hoc* test: Dunn-Bonferroni; error bar: 95% CI; *P<0.05, pairwise comparisons of other groups: P < 0.01).

Hematoxylin and eosin (HE) staining showed that the morphological structure of the sham group was intact, with well-demarcated gray and white matter, homogeneous neuronal distributions, well-arranged nerve fibers and intercellular structures, and well-defined nuclei (via Nissl staining). At one week after SCI, the structure of gray and white matter was not integrated, vacuoles and vesicles were found in the injured area, axons were disordered, and neurons were missing. Nissl staining showed a decrease in the number of neurons within gray matter. In the OEC+NSC group, HE staining showed reduced syringomyelia and Nissl staining showed an increased number of neurons compared to these parameters in the DMEM group (Figure 4). Collectively, these results indicate that OEC/NSC co-transplantation reduced the formation of scar tissue and loss of neurons, and protected against tissue damage.

### Locomotor function in SCI rats after cell transplantation

**Figure 5:**
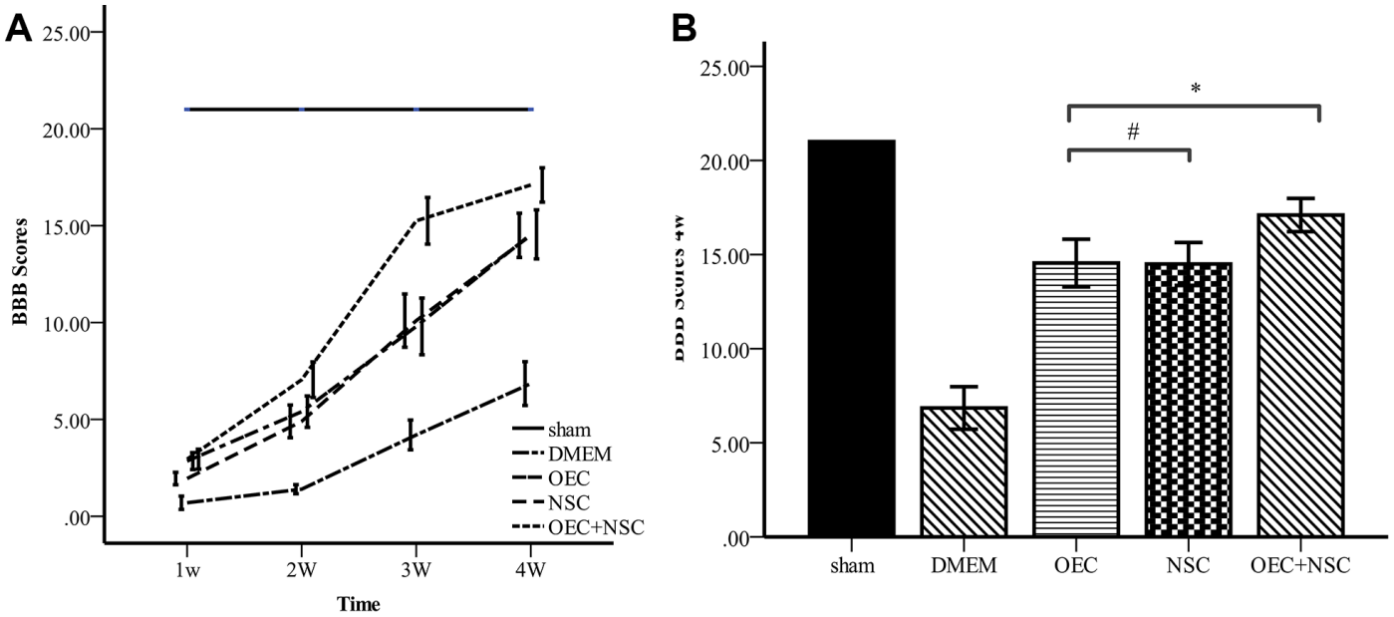
**Comparison of BBB scores.** (**A**) BBB scores for each group varied over time. Error bar: 95% CI. (**B**) BBB scores at four weeks after cell transplantations. (mean ± SEM; two-way ANOVA, *post-hoc* test: Dunn-Bonferroni; error bar: 95% CI; **P<0.01).

To determine the therapeutic effects of cell transplantation, we used scores from the Basso, Beattie, and Bresnahan (BBB) 21-point open-field locomotor rating scale to evaluate motor function (Table 1, Figure 5). All rats attained a BBB score of 21 before the injury was induced, and no significant differences were observed between the five groups at this time. In contrast, BBB scores were reduced to 0–1 immediately after induction of SCI in the four groups receiving SCI, whereas BBB scores in the sham group remained stable. After cell transplantation, the BBB scores of each treatment SCI group increased over time, and the OEC+NSC group showed the most significant improvement among these groups. Compared with those of the DMEM group (0.7±0.73), BBB scores in all cell-transplantation groups were significantly improved at one week after transplantations (P=0.000). Compared with those in the NSC group (1.9±0.69), BBB scores were significantly improved the OEC group (2.85±0.93, P=0.000) and OEC+NSC group (2.95±1.10, P=0.000); in contrast, there was no significant difference in this parameter between the OEC group and the OEC+NSC group (P=1.000). At 2–4 weeks after transplantation, the BBB scores of each group continued to improve. Compared with those of the NSC group (14.5±2.44), the BBB scores of the OEC group showed no statistical difference, they were not significantly different (14.55±2.70, P=0.000). However, the BBB scores of the OEC+NSC group (17.1±1.39) were significantly improved compared with those of the NSC group and OEC group (vs NSC group, P=0.006; vs OEC group, P=0.043).

**Table 1:** BBB scores.

	**sham**	**DMEM**	**OEC**	**NSC**	**OEC+NSC**
1W	21±0.00	0.7±0.73	2.85±0.93	1.95±0.69	2.95±1.10
2W	21±0.00	1.4±0.50	5.4±1.73	4.9±1.80	7.05±1.96
3W	21±0.00	4.2±1.64	9.8±3.12	10.1±2.94	15.252.57
4W	21±0.00	6.85±2.41	14.55±2.70	14.5±2.44	17.1±1.39

One week after surgery, there was no statistically significant difference in BBB score between OEC group OEC+NSC group (P=1); 2W, there was no significant difference between the OEC group and the NSC group and the OEC+NSC group. At 3W and 4w, the difference between the OEC group and the NSC group was not statistically significant, and pairwise comparison between the other groups at all 4 time points was statistically significant (p value < 0.01).

### SSRs in rats

**Figure 6:**
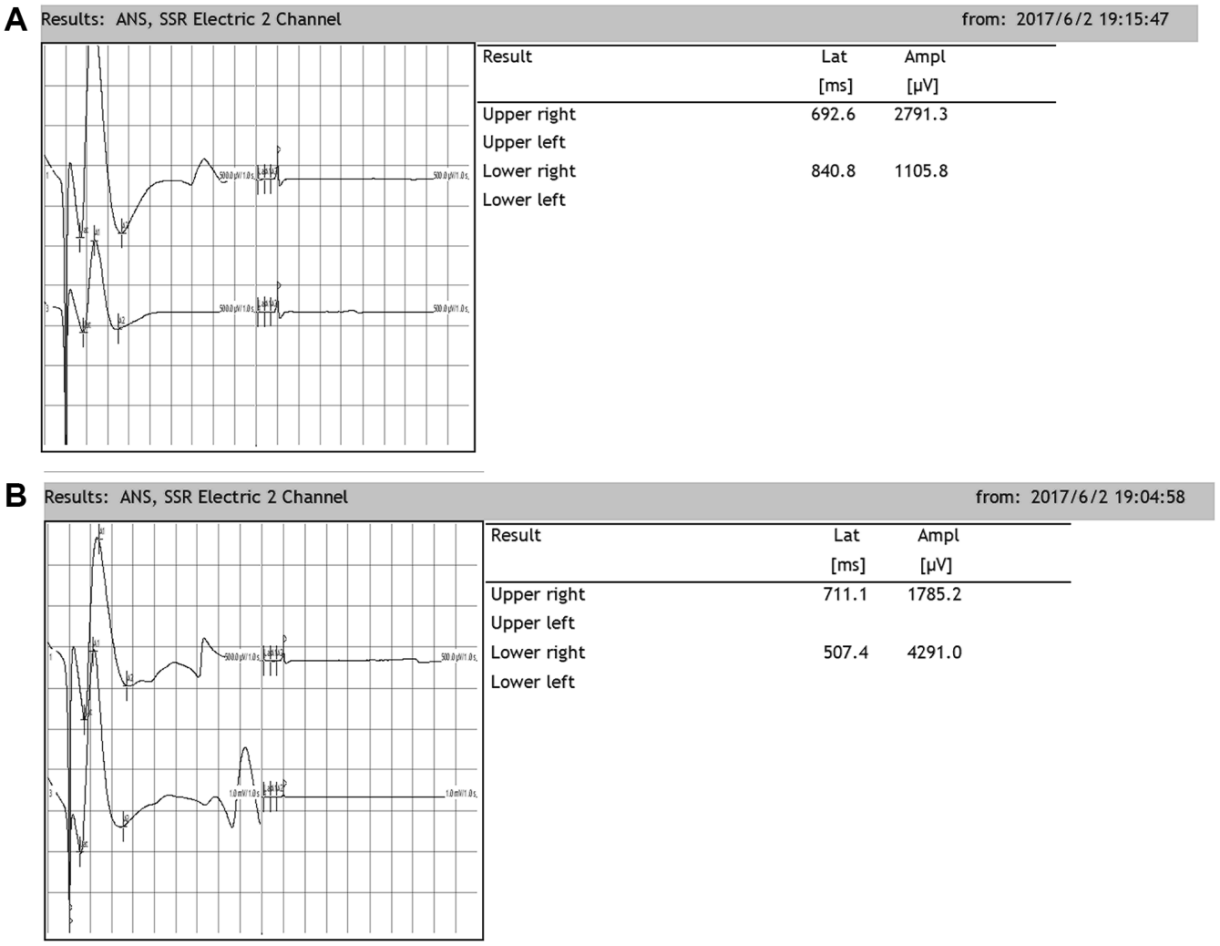
**Representative sympathetic skin responses in normal rats.** (**A**) We stimulated the left median nerve and recorded in the upper-right or lower-right limbs. (**B**) We stimulated the left tibial nerve and recorded in the upper-right or lower-right limb.

Rats exhibit SSRs that can be determined by electromyography. The resulting SSR waveforms are similar to those observed in humans, consisting of a biphasic or triphasic wave (although the biphasic wave occurs most frequently). Generally, an SSR consists of a low-amplitude negative wave followed by a high-amplitude positive wave. In our present study, when the median nerve was stimulated, SSRs were elicited from both the upper and lower limbs. There was no significant difference in the amplitudes or latencies between the two upper or lower limbs. However, there were significant differences in the latencies and amplitudes between the upper and lower limbs, which were similar when the tibial nerve was stimulated (Figure 6).

### Changes in SSRs in SCI rats after cell transplantation

**Figure 7:**
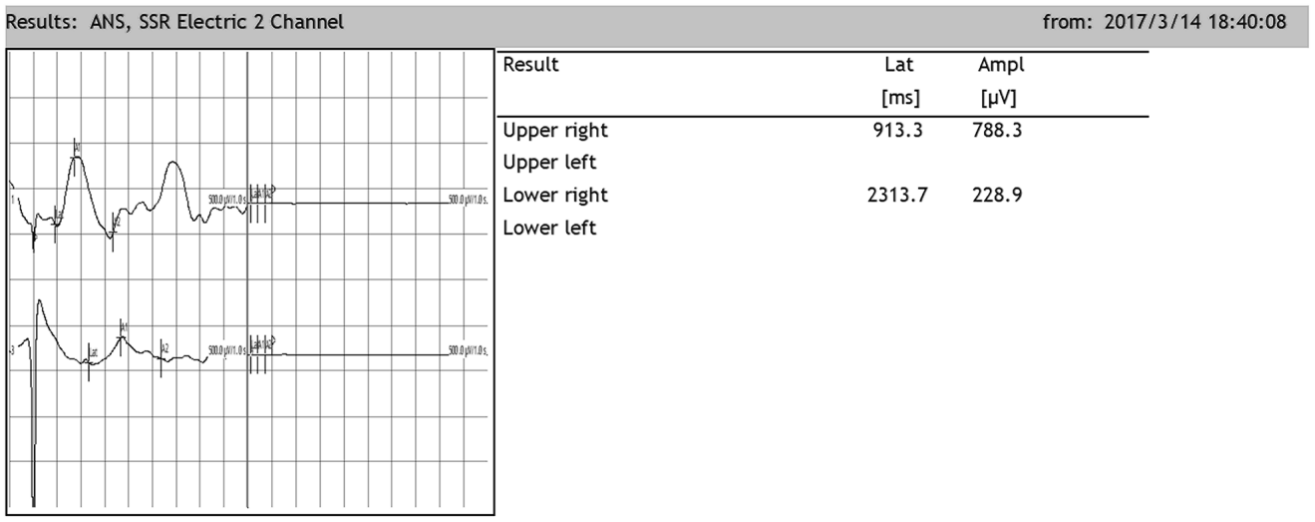
**Representative sympathetic skin responses in SCI rats.** The median nerve was stimulated at the left wrist, and the recording points were located in the right palm of the forepaw and the right palm of the hindpaw.

**Figure 8:**
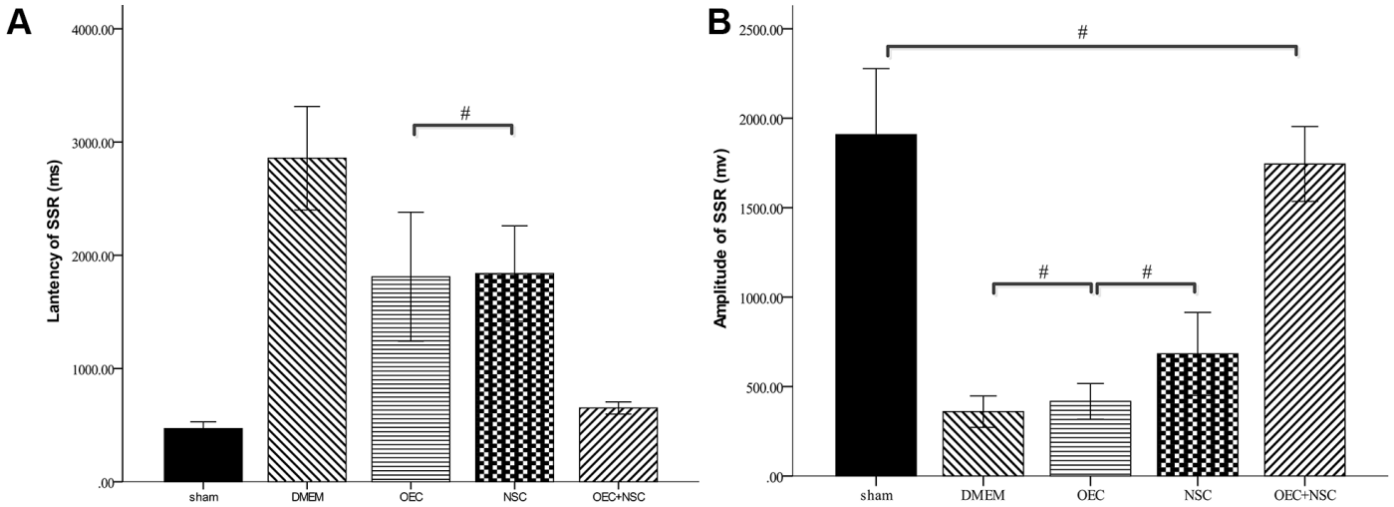
**Sympathetic skin responses in SCI rats after cell transplantations.** ^#^P>0.05, pairwise comparisons of other groups yielded P values < 0.01 (mean ± SEM; one-way ANOVA, *post-hoc*: Dunn-Bonferroni; Error bar: 95% CI).

After SCI, the SSR ejection rate decreased (DMEM group: 8/20, OEC group: 13/20, NSC group: 12/20, OEC+NSC group: 18/20). When SSRs were induced, their amplitudes were reduced and their latencies were prolonged following SCI (Figure 7). As can be seen from Table 2, changes caused by SCI in all the cell-transplantation groups were reduced after two weeks of treatment. There were no statistically significant differences in SSR parameters between the OEC (latency: 1810.72±942.96 ms; amplitude: 417.25±164.82 μV) and NSC transplantation groups (latency: 1755.58±987.95 ms; amplitude: 682.8±367.84 μV), but there were significant differences in both the SSR latencies and amplitudes between the OEC+NSC group (latency: 651.62±108.08 ms, P=0.000; amplitude: 2077.66±778.04 μV, P=0.000) and the single-transplantation groups (Table 2, Figure 8).

**Table 2:** Quantitative SSR parameters in SCI rats after treatment.

	**Sham**	**DMEM**	**OEC**	**NSC**	**OEC+NSC**
Latency	469.52±129.54	2857.63±546.54	1810.72±942.96#	1838.92±662.78#	651.62±108.08
Amplitude	1908.39±788.65#	359.75±104.92#	417.25±164.82#	682.8±364.87	1744.33±420.73#
Positive rates	20/20	8/20	13/20	12/20	18/20

There was no significant difference in latency between the OEC and NSC groups, #P=1.00, Pairwise comparison of other groups p value < 0.01(means ± SEM; one-way ANOVA, post-hoc:Dunn-Bonferroni; Error bar: 95% CI); There was no significant difference in amplitude between the OEC+NSC group and sham group and between the DMEM group, OEC group and the OEC group (P=1.00), Pairwise comparison of other groups p value < 0.01(means ± SEM; one-way ANOVA, post-hoc:Dunn-Bonferroni; Error bar: 95% CI).

## DISCUSSION

The present study elucidated the following. First, the SCI microenvironment led to necroptosis of grafted NSCs via RIP3/MLKL, thus inhibiting their survival and therapeutic efficacy in the spinal cord. Second, transplanted OECs counteracted SCI-induced necroptosis, protecting co-transplanted NSCs and prolonging their intramedullary survival to promote nerve regeneration. Third, intraspinal transplantation of NSCs and OECs after SCI effectively mitigated autonomic neurological dysfunction. Fourth, we demonstrated that SSRs of SCI rats were elicitability decreased, and that remaining SSRs exhibited prolonged latencies and decreased amplitudes. Finally, we revealed that OEC/NSC co-transplantation not only improved motor function but also effectively alleviated autonomic nerve dysfunction.

Previous studies have shown that RIP3 and MLKL levels are up-regulated within 24 hours after SCI, reach a peak within three days, and remain up-regulated for 21 days [7]. Interestingly, the time course of this process is similar to that of secondary injury after SCI [20]. In the present study, we found that RIP3/MLKL levels and necroptosis were increased at two weeks after SCI. At this time after SCI, apoptosis has been shown to be rare [21], as it usually peaks at three days and nearly disappears by seven days after SCI. Hence, necrosis may play a more important role in secondary injury after SCI.

Many studies have suggested that necroptosis can occur in various cells within the nervous system, including neurons, astrocytes, and oligodendrocytes [22]. However, little attention has been paid to whether necroptosis affects transplanted cells. Thus, in in the present study, we used SCI homogenates to simulate the SCI microenvironment *in vitro*. We found that SCI homogenates up-regulated the expression levels of RIP3 and MLKL in naïve NSCs. This result suggests that the SCI microenvironment can cause necroptosis of uninjured cells. We also found that RIP3/MLKL- mediated necroptosis not only occurred in intrinsic cells within the spinal cord, but also in grafted NSCs. This may represent one of the main reasons why transplanted cells rarely survive and yield low efficacies after cell transplantation.

In our present study, MTT assays showed that OECs promoted the growth of NSCs under SCI conditions *in vitro*. Since we speculated that this phenomenon may have been due to OECs inhibiting necroptosis of NSCs, we then measured RIP3 and MLKL levels in NSCs cultured with SCI-h and/or OECs. The results confirmed our hypothesis that when NSCs were co-cultured with SCI-h and OECs, SCI-h-induced upregulation of RIP3 and MLKL was decreased, indicating that necroptosis was suppressed. To further corroborate this finding, we measured the expression levels of RIP3/MLKL in the spinal cords of SCI rats after cell transplantations via Western blotting and obtained similar results.

Furthermore, we sought to determine whether the protective effects of OECs on grafted NSCs could improve the therapeutic efficacy of cell transplantations. We observed HE and Nissl staining of spinal-cord tissue at two weeks after cell transplantations, and the result showed that OECs reduced the formation of cavities and loss of neurons following SCI. These findings suggest that OEC transplantation can prevent necroptosis of different cell types within the spinal cord, thus providing a neuroprotective effect and improving various pathological conditions after SCI.

## CONCLUSION

Our present findings suggest that co-grafting of NSCs and OECs ameliorates SCI, possibly by inhibiting RIP3/MLKL-mediated necroptosis and promoting the survival and proliferation of NSCs in the medulla. Our present findings also demonstrate that SSRs can be used to assess autonomic nerve function in rats. We found that SSR frequencies and amplitudes were decreased following SCI, whereas SSR latencies were prolonged, and that NSC/OEC co-transplantation ameliorated these SCI-induced changes in SSR parameters.

## MATERIALS AND METHODS

### Animals

All animal procedures were approved by the Animal Care and Ethics Committee of the University of Shandong First Medical University and were conducted in accordance with the National Institutes of Health guidelines. Experiments were performed on 100 adult Sprague-Dawley rats. All rats were housed under standard conditions with food and water provided *ad libitum*. One-hundred adult Sprague-Dawley rats, 10–12 weeks of age (200–250 g), received cell transplants after a lumbar-thoracic spinal cord transection and were maintained for one-week post-injury. Ten 24-h postnatal rat pups were used for harvesting and culturing of OECs and NSCs.

### Isolation and culturing of primary rat OECs

OECs were generated from newborn Sprague-Dawley rats. Ten postnatal rat pups were deeply anaesthetized with 10% chloral hydrate (0.3 ml/100 g, interperitoneally) and were sacrificed by cervical dislocation. OECs were generated from the olfactory bulbs, as previously described [23, 24]. After OEC dissection from the first two layers of the olfactory bulb, meninges and blood vessels were removed to reduce fibroblasts. The granular layer was washed twice with phosphate-buffered saline (PBS). The tissue was cut with an ophthalmic scissor and was digested with accutase (Sigma-Aldrich, St. Louis, MO, USA), after which it was placed in a 37° C incubator for 15 min. Subsequently, the tissue was centrifuged at 1,000 rpm/min for 5 min. The pellet was re-suspended in Dulbecco’s modified Eagle’s medium (DMEM)/F12 medium (Sigma-Aldrich; Merck KGaA). Finally, single-cell suspensions were produced using DMEM/F12 medium containing 20% fetal calf serum (FCS; Sigma-Aldrich, Merck KGaA), seeded in plastic culture flasks and cultured in an incubator at 37° C and 5% CO_2_. The media were changed every 2–3 days, with 50% of the medium replaced. OECs were stained with antibodies against p75 and S-100.

### Preparation of NSCs from rats

NSCs were extracted from the hippocampi of newborn rats within 24 h. After soaking in 75% alcohol, the newborn rats were decapitated to expose both hemispheres of the brain. The cortices were lifted to expose the hippocampi. The hippocampi were separated by blunt dissection and placed in DMEM/ F12 medium (Gibco, Paisley, Scotland, UK). Then the hippocampi were digested in accutase (Sigma-Aldrich, St. Louis, MO, USA) and centrifugation was performed at 800 rpm/min for 5 min. Cells were resuspended with DMEM/12. Cell-count plates were used to count the number of cells, and the number of cells was adjusted to 1*10^6^ cells/L. These cells were then placed in a culture flask and were cultured in fresh DMEM/F12 (1:1) containing 20 ng/mL basic fibroblast growth factor, 20 ng/mL epidermal growth factor, 2% B27, 1% N2 (all from Abcam, Cambridge, UK), 100,000 U/L penicillin (Sigma-Aldrich), and 10,000 U/L streptomycin [25]. The cultures were kept in an incubator at 37° C and 5% CO_2_. Mouse anti-nestin (1:200, Abcam) was used to identify the purity of NSCs. NSCs were digested into single cells by trypsin and then cell smears were prepared. After the smear was completely dried, immunofluorescent staining was performed. The numbers of DAPI^+^ and DAPI^+^/nestin^+^ cells were counted under a microscope, and the ratio was indicative of the positive survival rate of NSCs. The results indicated that more than 90% of cells were nestin-positive, indicating a fairly high purity of NSCs. The cell suspension was collected and adjusted to a concentration of 1.0×10^5^ cells/L for transplantation. NSCs were labeled with an RFP lentivirus.

### Effects of OECs on proliferation of NSCs

### Preparation of OEC-CM

After purification, OECs were stably cultured for three passages, the old medium was discarded, the culture bottle was washed twice with serum-free DMEM/F12 medium, and the fresh serum-free DMEM/F12 medium was added again. OECs were cultured according to the aforementioned procedure (in subsection 5.2) above for 3 d. The upper suspension was centrifuged for 10 min at 3,000 rpm/min at 4° C, and the supernatant was collected and filtered through a 400-mush sift strainer to remove debris. The supernatant was then mixed as the OEC-CM and was frozen at −80° C until further use, as previously described [26].

### Preparation of spinal cord homogenates

Three days after SCI, rats were perfused with ice-cold PBS, and a 5-mm long segment of the injured spinal cord centered on the injury epicenter was removed and rapidly frozen on dry ice. For naïve rats, the same region of the spinal cord as that of the injured rats was removed for this preparation. Spinal cords of five rats for each group were pooled together in 1 mL of DMEM/F12. The tissue was homogenized by using pestles for 2 min on ice. The homogenate was cleared by centrifugation at 12,000 rpm for 15 min, and the total protein concentrations of cleared supernatants were measured via BCA assays. After adjusting the total protein concentration of each sample, the aliquots were kept at −80° C until further use [27, 28].

After OECs were cultured for 7 d, the cells were passaged. The old medium was discarded and the culture bottle was washed twice with serum-free DMEM/F12. After digestion for 15 min by accutase at 37° C, the supernatant was removed, and then serum-free DMEM/F12 was added. Single-cell suspensions were prepared by repeated blow-beating with a polished Babel straw. The cell density was adjusted to 1×10^8^ cells/L.

### Effects of OECs on proliferation of NSCs

NSCs were cultured for 7 d and the cell density was adjusted to 1× 10^8^ cells/L. Cells were divided into three groups as follows: OEC group: 2 mL of OEC suspension and 2 mL of NSC suspension; OEC-CM group: 2 mL of OEC-CM and 4 mL of NSC suspension; and sham group: 2 mL of complete medium and 4 mL of NSC suspension. Cell proliferation was measured by an MTT assay at 1, 3, 5, and 7 d after being cultured.

### Effects of OECs on proliferation of NSCs in the SCI microenvironment

NSCs were cultured for 7 d and the cell density was adjusted to 1× 10^8^ cells/L. Cells were divided into the following four groups: naïve-h group: 4 mL NSC suspension and 2 mL naïve-h; SCI-h group: 4 mL NSC suspension and 2 mL SCI-h; naïve-h+OEC group: 2 mL NSC suspension, 2 mL naïve-h, and 2 mL OEC suspension; SCI-h+OEC group: 2 mL NSC suspension, 2 mL SCI-h and 2 mL OEC suspension; and vehicle group: 4 mL NSC suspension + 2 mL complete medium.

### Animal groupings, SCI model, and cell transplantation

The rats were divided into a sham group (no SCI + no cell transplantation), DMEM group (SCI + DMEM), OEC group (SCI + OEC transplantation), NSC group (SCI + NSC transplantation), and OEC + NSC group (SCI + OEC transplantation + NSC transplantation).

Healthy adult rats (n=100) were anesthetized with 10% chloral hydrate via the abdominal cavity. After the anesthesia took effect, each rat was fixed on its stomach, its skin was disinfected, and a median ridge incision (about 3 cm) was made. The T9–11 spinous process and vertebral lamina were exposed, excised, and the T4 segment medulla was exposed. With a 10-g weight, the modified Allen's impactor free falling from 25 cm caused acute SCI in rats, and the weight remained in its terminal position for 3 min after impact.

Criteria for successful SCI modeling were as follows: engorged and thickened dural veins; local purplish-red color; spasmodic swinging of the tail; and hind-limb retrusion. In the sham group, only the T9–11 laminectomy was performed without damage to the spinal cord.

Cell transplantation was performed immediately after SCI. Specifically, 5 μL of cell suspension (approximately 1.0×10^5^ cells/L) or DMEM/F12 was slowly injected into the upper and lower ends of the injury subarea with a microsyringe, after which the wound was closed layer by layer. Penicillin was given two times a day for 3 d to prevent infection. Micturition was assisted twice a day until a micturition reflex was established in each rat.

### Evaluation of hindlimb movements

The BBB 21-point open-field locomotor rating scale was used for evaluating hindlimb movements. Rats were placed on a circular platform with a diameter of 2 m. Walking and hindlimb movement scores were observed and recorded. In the first stage (0–7 points), joint movement of the hindlimb was scored. In the second stage (8–13), scores were judged according to gait and hindlimb coordination. In the third stage (14–21 points), points were scored based on the movement of the claws. The three stages comprised a total of 21 min. Rats were evaluated at one, two, three, and four weeks after transplantation.

### Evaluation of autonomic function

Autonomic function was evaluated by SSRs. SSRs were recorded by the Keypoint-4 Electromyography Evoked Potential System (Medtronic; Minneapolis, Minnesota, USA). SSRs were determined for both upper limbs and lower limbs and were evoked by electrical stimulation (0.2-ms duration, 1-Hz frequency, 0.5-mV/D sensitivity). The recording electrodes were placed on the plantar surface of each foot and the palm of each hand, and the references were placed on the dorsum of the foot and hand. The tibial nerve was stimulated using a saddle electrode in the medial malleolus, while the median nerve was stimulated in the midline of the wrist using the same electrode. The stimulus intensity was initially 40 mA, and was then increased to 60 mA. All rats were administrated four stimuli until a stable waveform was observed. The interval between stimuli occurred at random intervals of more than 1 min each to avoid habituation. The SSR latencies and amplitudes were recorded at 1 d before transplantation, as well as at four weeks after transplantation.

### Detection of NF200, RIP3, and MLKL via western blotting

Rats in each group were sacrificed on an ice bath at four weeks after operations. Protein was extracted from the injured central spinal cord and the same segment was obtained in the sham group. Protein levels of NF200, RIP3, and MLKL were determined by Western blotting.

### Histopathological assessments

The rats were intraperitoneally anesthetized with 10% chloral hydrate, fixed via routine perfusion, and T3–T5 spinal cord segments were exposed and quickly harvested. After fixation with 4% paraformaldehyde for 24 h, tissues were subjected to gradient sucrose dehydration, OCT embedding, and were preserved at −80° C. HE staining and Nissl staining were performed on 15-μm-thick coronal sections.

### Immunofluorescent staining

Frozen sections were fixed with 4% PFA for 15 min, washed three times with PBS, permeabilized with 0.5 triton X-100 for 20 min, and washed three times with PBS. The sections were blocked with 5% goat albumin for 60 min and incubated with the appropriate primary antibody overnight at 4° C. The following primary antibodies were used for immunofluorescent staining: rat anti-P75 NGF (1:200; Abcam, Cambridge, UK), goat anti-S-100 (1:200; Abcam), and rat anti-nestin (1:200; Abcam). FITC/CY3-labeled fluorescent secondary antibody (1:200; Abcam) was incubated for 2 h. Finally, DAPI was used for staining cellular nuclei.

### Statistical analysis

Statistical analysis was conducted using SPSS 19.0 software (SPSS Ins, Chicago, IL, USA). All continuous variables were first examined by Shapiro-Wilk tests to determine if they exhibited normal distributions. For normal continuous variables, we used the mean ± standard deviation to report descriptive statistics. Levene's tests were used to assess the assumption of homogeneity of variances for these variables. Two-way repeated-measures analysis of variance (ANOVA) was applied to assess differences in repeated measures at different time points. One-way ANOVA was performed to assess significant differences among different groups and the positive rates were tested by Chi-square tests. Dunn-Bonferroni *post-hoc* tests were used for all pairwise comparisons. Chi-Square tests were conducted to assess statistical differences in categorical variables. P values less than 0.05 were considered statistically significant via two-tailed tests.
